# Structure determines function—the role of topology in the functionality of gene circuits

**DOI:** 10.1093/synbio/ysaa008

**Published:** 2020-06-19

**Authors:** Daniel Bojar

**Affiliations:** y1 Wyss Institute for Biologically Inspired Engineering, Harvard University, Boston, MA, USA; y2 Department of Biological Engineering and Institute for Medical Engineering & Science, Massachusetts Institute of Technology, Cambridge, MA, USA

**Keywords:** synthetic biology, gene circuit

As synthetic biologists, we sometimes forget the toggle switch and the self-activating switch, the foundational advances that launched the entire field of synthetic biology a mere two decades ago. As the first in a long line of increasingly sophisticated gene circuits with applications in biocomputing and biomedical therapies, these combinations of genetic parts in the humble bacterium *Escherichia coli* demonstrated that biology can—in principle—be programmed. In a recent study in the journal *Nature Chemical Biology* (1), Zhang *et al.* from the group of Xiao-Jun Tian at Arizona State University revisited the toggle switch and the self-activating switch, demonstrating the differential impact of cell division and growth on the function of these circuits, which could make some circuit designs unviable in the environment of dilution or growth characterizing many applications.

As the output of the self-activating switch activates its own transcription, it should exhibit a stable ON-state beyond a certain inducer threshold. Yet what Zhang *et al.* discovered was that, once these green fluorescent protein (GFP)-positive, ON-state bacteria are diluted, the formerly stable ON-state disintegrates and the bacteria were suddenly indistinguishable from those that were never stimulated in the first place. Theory and their mathematical models, however, predicted that these once-ON-bacteria would remain ON, even after dilution. The supposedly stable memory of the self-activating switch was broken by a simple dilution.

The hidden variable that accounted for the circuit’s memory lapse was growth. It has been recently appreciated that gene circuits place a metabolic burden on cells and therefore inhibit growth (2), while Zhang *et al.* additionally discovered that growth inhibited the functionality of their gene circuit. This resulted in a seesaw dynamic after diluting cells into medium rich with inducer: first, GFP fluorescence crashed, and then, after cell growth subsided, GFP resumed production. Factoring in the interfacing of growth and gene circuit into their models indeed resolved any unexplained differences in circuit memory. Interestingly, dilution into conditioned rather than fresh medium, thus inhibiting rapid growth, did preserve the memory of the self-activating switch.

Naturally, Zhang *et al.* investigated whether this growth feedback also affected other circuit architectures, such as the toggle switch that can be used to switch between two stable states. Yet, overall, the toggle switch seemed to exhibit a much broader resistance to memory loss through growth effects, as long as the two constituting transcription factors operated on a similar timescale. Dilution into fresh or conditioned medium led to nearly the same output in terms of GFP fluorescence, demonstrating the perseverance of memory. The authors also note the crucial difference between transcriptional activation (self-activating switch) and inhibition (toggle switch), as the former seemed much more sensitive to absolute levels of transcription factor, while the latter operated more akin to a threshold. Thus, the inherent wiring or topology of the circuit affects its robustness to growth feedback and its resulting functionality.

The findings of Zhang *et al.* could have important implications for applications of these circuits and it will be crucial to ascertain where and to what extent these circuit properties affect their functionality. For example, there is increasing interest in homing genetically engineered bacteria to the gut microbiome. Consequences of recurring dilution after food ingestion will have to be factored into circuits designed for the gut. While the authors did not examine this particular application, it would be interesting to explore whether intrinsically robust circuit topologies can be designed based on the knowledge of how circuits interact with growth.

While these effects have been demonstrated in *E. coli*, the generality of these findings in other synthetic biology model organisms should also be investigated to gauge their relevance. Nonetheless, circuit design rules or guidelines could be derived from this work and further work needs to be conducted to elucidate whether inhibition provides an inherently more robust tool for circuit design than activation. What studies such as this one clearly demonstrate, is that even after two decades we can still uncover fundamental properties of these foundational building blocks of synthetic biology?

*Conflict of interest statement*. None declared.
